# A possible link between recurrent upper respiratory tract infections and lower cytokine production in patients with Q fever fatigue syndrome

**DOI:** 10.1002/eji.201848012

**Published:** 2019-05-09

**Authors:** Ruud P.H. Raijmakers, Anne F.M. Jansen, Stephan P. Keijmel, Jos W.M. van der Meer, Leo A.B. Joosten, Mihai G. Netea, Chantal P. Bleeker‐Rovers

**Affiliations:** ^1^ Radboud Expertise Center for Q fever Department of Internal Medicine Division of Infectious Diseases Radboud University Medical Center Nijmegen The Netherlands; ^2^ Department of Internal Medicine Radboud University Medical Center Nijmegen The Netherlands; ^3^ Radboud Center for Infectious Diseases Radboud University Medical Center Nijmegen The Netherlands

**Keywords:** epigenetics, innate immune memory, Q fever fatigue syndrome, trained immunity, upper respiratory tract infections

## Abstract

Besides fatigue, many Q fever fatigue syndrome (QFS) patients also complain of frequently recurring upper respiratory tract infections with severe symptoms. We investigated whether immunologic dysregulation contributes to these complaints. Cytokine and chemokine production was measured after stimulating monocytes of QFS patients and age‐ and sex‐matched healthy controls with LPS and several viral ligands. The H3K4me3 mark of open chromatin was measured at the promoter regions of cytokines and chemokines that differed significantly from healthy controls. Monocytes of QFS patients produced significantly less TNF‐α (*p* = 0.032), IL‐1β (0.004, 0.024, and 0.008), IL‐6 (0.043), RANTES (0.033), IP‐10 (0.049), MCP‐1 (0.022), IL‐ 13 (0.029), and IL‐10 (0.026) than healthy controls when stimulated with various ligands. H3K4me3 expression was significantly lower in QFS patients than in healthy controls on the promoter regions of IL‐1β (*p* = 0.004), MCP‐1 (<0.001 and <0.001), IP‐10 (<0.001), IL‐10 (0.041), and IL‐13 (<0.001, <0.001, and 0.001). QFS patients showed diminished cytokine responses to various stimuli compared to age‐ and sex‐matched healthy controls, likely due to epigenetic remodeling and long‐term memory as a result from the acute Q fever infection. This might explain the upper respiratory tract ailments in QFS.

## Graphical abstract

Circulating monocytes of Q fever fatigue syndrome patients with recurrent upper respiratory tract infections produce less cytokines compared to those of healthy controls when stimulated with viral ligands, possibly due to epigenetic remodeling following the acute Q fever infection.

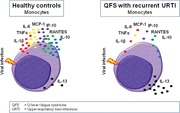

## Introduction

Q fever is a zoonosis caused by the intracellular Gram‐negative bacterium *Coxiella burnetii* 1. Initial infection with *C. burnetii* remains asymptomatic in around 60% of cases, but can also manifest as symptomatic disease, acute Q fever, usually manifesting as a flu‐like illness that is sometimes accompanied by hepatitis or pneumonia 1, 2. The Netherlands experienced the largest Q fever outbreak ever reported between 2007 and 2011. It is estimated that at least 32 200 individuals were infected, and around 4000 patients notified having a symptomatic acute Q fever infection 3. During an acute Q fever infection, the *C. burnetii* bacterium resides inside the phagolysosomes of monocytes and macrophages 1, 2. In order to survive and replicate in the phagolysosome, or *Coxiella* containing vacuole, of these cells, *C. burnetii* is known to alter and subvert various host cell processes 4, 5.

Q fever fatigue syndrome (QFS) is a debilitating post‐infective fatigue syndrome developing in approximately 20% of patients with an acute Q fever infection 6. It is characterized by complaints of fatigue that last for at least 6 months, often coinciding with complaints of musculoskeletal pain, neurocognitive problems, sleeping problems, headache, (night) sweating, and mood disorders 7. Many patients with QFS also complain of frequently recurring and severe upper respiratory tracts infections. Although the exact etiology of QFS remains unclear, results from previous studies and the nature of concomitant complaints are compatible with an inflammatory component in its pathophysiology 8, 9.

Recently, the dogma that innate immune cells, e.g. monocytes and macrophages, are unable to acquire immunologic memory, was challenged 10. It was shown that monocytes and likely also myeloid progenitor cells are able to acquire long‐lasting immunologic memory (also termed “trained immunity”) through epigenetic remodeling at the promoter regions of various cytokines 11, 12. Depending on their initial challenge, these cells show altered (decreased or increased) responses to secondary challenges such as infections 13, 14. Given these findings, it could be hypothesized that QFS patients who experience more frequent and severe upper respiratory tract infections since their acute Q fever infection, exhibit epigenetic remodeling of chromatin at the level of immune genes involved in host defense against viral upper respiratory tract infections. This remodeling could then result in altered cytokine responses to such infections. We test this hypothesis by conducting in vitro stimulation experiments on monocyte responsiveness of QFS patients with frequently recurring and severe upper respiratory tracts infections, together with an investigation of epigenetic changes at various cytokine promoter regions of these monocytes.

## Results

### Patients and controls

QFS patients had a median age of 47 (interquartile range, IQR, 41–54) and predominantly female distribution (66.67%). All QFS patients had IgG phase I or phase II titres ≥ 1:16, but IgG phase I ≤ 1:512, and none of them showed serological signs of an acute or recent Q fever infection, reflected by IgM antibodies in absence of IgG antibodies. All healthy controls had negative IgM phase I and II, and IgG phase I and II titres.

### Lower cytokine responses of QFS patients compared to healthy controls

**Figure 1:**
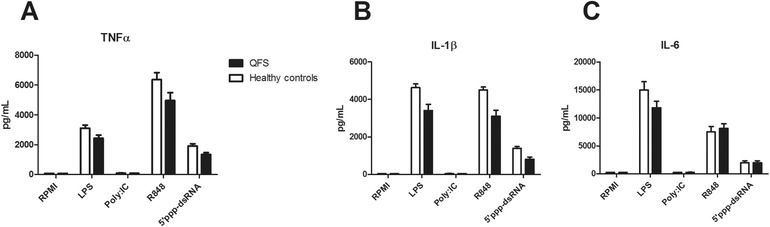
Production of TNF‐α, IL‐1β, and IL‐6 in QFS patients compared to healthy controls, Monocytes were stimulated with RPMI, LPS, Poly I:C, R848, or 5’ppp‐dsRNA for 24 h. (A) Monocytes of QFS patients, stimulated with 5’ppp‐dsRNA, produced significantly less TNF‐α (*p* = 0.032) than those of healthy controls. (B) Monocytes of QFS patients, stimulated with LPS, R848, and 5’ppp‐dsRNA, produced significantly less IL‐1β (*p* = 0.008, *p* = 0.004, and *p* = 0.024, respectively) than those of healthy controls. (C) Monocytes of QFS patients, stimulated with LPS, Poly I:C, R848, and 5’ppp‐dsRNA, show no significant difference in IL‐6 production compared to those of healthy controls. Cytokines were measured with ELISA. Data were analyzed with the Mann‐Whitney test and are depicted as mean ± SEM. Data are derived from one single experiment that consisted of 15 patients and 11 healthy controls. Abbreviations: QFS, Q fever fatigue syndrome; RPMI, Roswell Park Memorial Institute culture medium; PolyI:C, R848, and 5’ppp‐dsRNA, various viral ligands. *p ≤ 0.05; **p ≤ 0.01

Monocytes of QFS patients produced significantly less TNF‐α when stimulated with 5’ppp‐dsRNA (*p* = 0.032) than those of healthy controls (Fig. 1A). This was also the case for IL‐1β when stimulated with R848 (*p* = 0.004), 5’ppp‐dsRNA (*p* = 0.024), and LPS (*p* = 0.008; Fig. 1B). No significant differences were found for IL‐6 (Fig. 1C).

**Figure 2:**
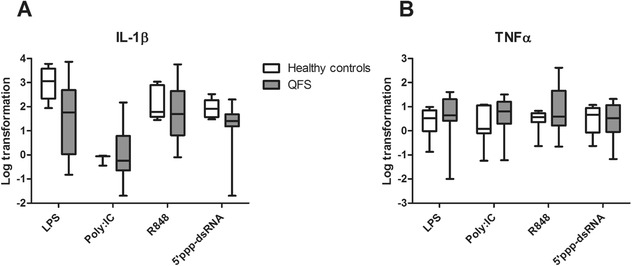
mRNA expression of IL‐β and TNF‐α in QFS patients compared to healthy controls, Monocytes were stimulated with LPS, Poly I:C, R848, or 5’ppp‐dsRNA for 24 hours. (A) Monocytes of QFS patients showed significantly decreased mRNA expression of IL‐1β compared to healthy controls when stimulated with 5’ppp‐dsRNA (*p* = 0.036). (B) Monocytes of QFS patients show no significantly difference in mRNA expression of TNF‐α compared to healthy controls. mRNA expression was measured with qPCR. The following primers were used: TNFα FWD‐5’‐AACGGAGCTGAACAATAGGC‐3’, REV‐5’‐TCTCGCCACTGAATAGTAGGG‐3’, IL‐1β FWD‐5’‐GCCCTAAACAGATGAAGTGCTC‐3’, REV‐5’‐GAACCAGCATCTTCCTCAG‐3’. RNA expression was corrected for differences in loading concentration using the signal of housekeeping protein GAPDH. Data were analyzed with the Mann–Whitney test and are depicted as mean ± SEM. Data are derived from one single experiment that consisted of 13 patients and seven healthy controls. Abbreviations: QFS, Q fever fatigue syndrome; Poly I:C, R848, and 5’ppp‐dsRNA, various viral ligands; RPMI, Roswell Park Memorial Institute culture medium; qPCR, quantitative real‐time PCR. *p ≤ 0.05; **p ≤ 0.01

By measuring expression of TNFα and IL‐1β mRNA, we investigated whether the changes in cytokine levels were regulated pretranscriptionally, transcriptionally, or posttranscriptionally. A similar trend for IL‐1β and TNF‐α was observed in qPCR data (Fig. 2A and B), showing significantly decreased IL‐1β mRNA expression in monocytes of QFS patients compared to those of healthy controls, when stimulated with 5’ppp‐dsRNA (*p* = 0.036).

**Figure 3:**
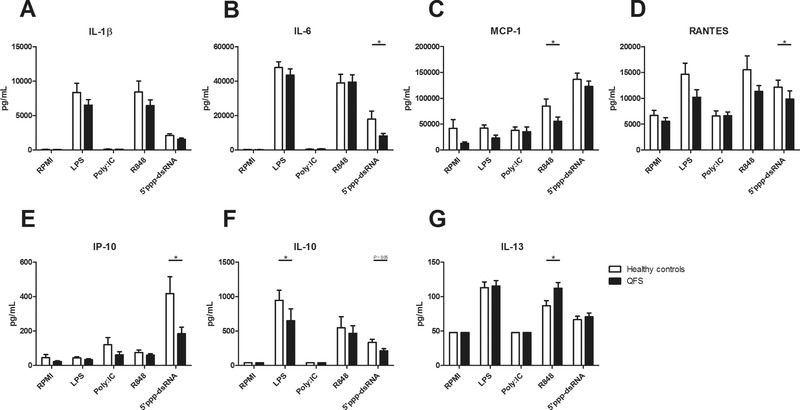
Production of IL‐1β, IL‐6, MCP‐1, RANTES, IP‐10, IL‐10, and IL‐13 in QFS patients compared to healthy controls, Monocytes were exposed to RPMI, LPS, Poly I:C, R848, or 5’ppp‐dsRNA for 24 h. (A) Monocytes of QFS patients, stimulated with LPS, Poly I:C, R848, and 5’ppp‐dsRNA, showed no significant difference in IL‐1β production compared to those of healthy controls. (B) Monocytes of QFS patients, stimulated with 5’ppp‐dsRNA, produced significantly less IL‐6 (*p* = 0.043) than those of healthy controls. (C) Monocytes of QFS patients, stimulated with R848, produced significantly less MCP‐1 (*p* = 0.022) than those of healthy controls. (D) Monocytes of QFS patients, stimulated with 5’ppp‐dsRNA, produced significantly less RANTES (*p* = 0.033) than those of healthy controls. (E) Monocytes of QFS patients, stimulated with 5’ppp‐dsRNA, produced significantly less IP‐10 (*p* = 0.049) than those of healthy controls. (F) Monocytes of QFS patients, stimulated with LPS, produced significantly less IL‐10 (*p* = 0.026) than those of healthy controls. (G) Monocytes of QFS patients, stimulated with R848, produced significantly more IL‐13 (*p* = 0.029) than those of healthy controls. Cytokines and chemokines were measured with luminex assay. Data were analyzed with the Mann–Whitney test and are depicted as mean ± SEM. Data are derived from one single experiment that consisted of 15 patients and 11 healthy controls. Abbreviations: MCP, monocyte chemoattractant protein; RANTES, regulated on activation, normal T cell expressed and secreted; RPMI, Roswell Park Memorial Institute culture medium; IP, interferon gamma‐induced protein; QFS, Q fever fatigue syndrome; Poly I:C, R848, and 5’ppp‐dsRNA, various viral ligands. *p ≤ 0.05

After this initial observation, a Luminex assay was performed for broader exploration of involved cytokines and chemokines. A significantly decreased production of IL‐6 (*p* = 0.043), RANTES (*p* = 0.033), and IP‐10 (*p* = 0.049) was found by monocytes of QFS patients compared to those of healthy controls when stimulated with 5’ppp‐dsRNA, together with a near significantly decreased production of IL‐10 (*p* = 0.056; Fig. 3B and D–F). This was also the case for MCP‐1 (*p* = 0.022) when stimulated with R848, and IL‐10 (*p* = 0.026) when stimulated with LPS (Fig. 3C and F). Monocytes of QFS patients produced more IL‐13 (*p* = 0.029) than those of healthy controls when stimulated with R848 (Fig. 3G). Contrary to ELISA, no significant differences in IL‐1β production were found with the Luminex assay (Fig. 3A).

### Histone modifications at cytokine promoter regions of QFS patients compared to healthy controls

**Figure 4:**
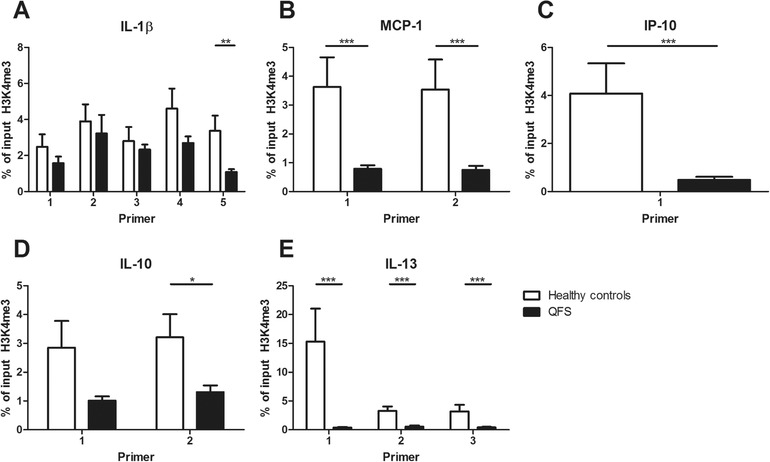
Expression of H3K4me3 in monocytes of QFS patients compared to healthy controls, Expression of H3K4me3 in monocytes of QFS patients was compared to healthy controls, using various primers. (A) Decreased expression of H3K4me3 on the IL‐1β promoter region, using primer 5 (*p* = 0.004). (B) Decreased expression of H3K4me3 on MCP‐1 promoter region, using primer 1 and 2 (*p* < 0.001 and *p *< 0.001, respectively). (C) Decreased expression of H3K4me3 on the IP‐10 promoter region, using primer 1 (*p* < 0.001). (D) Decreased expression of H3K4me3 on the IL‐10 promoter region, using primer 2 (*p* = 0.041), not primer 1 (*p* = 0.078). (E) Decreased expression of H3K4me3 on the IL‐13 promoter region, using primer 1, 2, and 3 (*p* < 0.001, *p <* 0.001, and *p* = 0.001, respectively). H3K4me3 expression was measured with qPCR. Primers used in the reaction are listed in Table S1. Samples were analyzed following a comparative Ct method, myoglobin was used as a negative control and H2B as a positive control for H3K4me3. Data were analyzed with the Mann–Whitney test and are depicted as mean ± SEM. Data are derived from one single experiment that consisted of 15 patients and 16 healthy controls. Abbreviations: H3K4me3, histone 3 lysine 4 trimethylation; MCP, monocyte chemoattractant protein; IP, interferon gamma‐induced protein; QFS, Q fever fatigue syndrome; qPCR, quantitative real‐time PCR. *p ≤ 0.05; **p ≤ 0.01; ***p ≤ 0.001

We investigated whether epigenetic markers on cytokine promoter regions were different between patients and controls. Histone modifications at cytokine promoter regions have been associated with trained immunity 11, especially histone 3 lysine 4 trimethylation (H3K4me3). At the promoter regions of IL‐1β, MCP‐1, IP‐10, IL‐10, and IL‐13, a decreased expression of H3K4me3 was found in QFS patients compared to healthy controls, for; IL‐1β (*p* = 0.004, primer 5), MCP‐1 (*p* < 0.001, primer 1 and *p* < 0.001, primer 2), IP‐10 (*p* < 0.001, primer 1), IL‐10 (*p* = 0.041, primer 2), and IL‐13 (*p* < 0.001, primer 1, *p* < 0.001, primer 2, and *p* = 0.001, primer 3; Fig. 4A–E). Negative and positive controls for H3K4me3 are depicted in Supporting Information Fig. 1.

## Discussion

In this study, we discovered that monocytes of QFS patients who experience more frequent and severe upper respiratory tract infections since their acute Q fever infection, had in general a decreased cytokine response to viral ligands and LPS. This decreased response was most likely regulated on a (pre‐)transcriptional level given the linked mRNA expression data for IL‐1β and, to a lesser extent, TNF‐α. In addition we demonstrated that there was a decreased expression of the open chromatin mark H3K4me3 on the promoter regions of these cytokines. It is conceivable that these low responses lead to greater susceptibility to viral infection in the upper airways.

The most common pathogens to induce upper respiratory tract infections are Rhinoviruses, Coronaviruses, and Influenza viruses 15. Most research on the role of antiviral cytokine and chemokine responses was done on Influenza. When looking at disease severity, human studies show consensus and indicate toward an association with increased cytokine and chemokine responses 16, 17. Studies on disease susceptibility are however scarce, mainly consist of mouse models, and show conflicting results. Both positive and negative associations are found when looking at disease severity, morbidity, and mortality in mice deficient for IL‐6 18 and IL‐1 19, 20, MCP‐1 21, and IP‐10 21 receptors. Nonetheless, it is conceivable that the decreased cytokine responses in our cohort of QFS patients contribute to an increased susceptibility to viral upper respiratory tract infections. Previous studies on cytokine responses in QFS found that white blood cells of QFS patients produce higher amounts of IL‐6 and IFN‐γ when exposed to *Coxiella* antigen 8, 9. One could therefore argue that immune regulation in QFS is ligand dependent, which makes it challenging to decipher its exact underlying immunopathologic mechanisms. It should be noted that this study solely included QFS patients who experience more frequent and severe upper respiratory tract infections since their acute Q fever while the above mentioned studies made no such distinction.

The finding that stimulation of primary monocytes from QFS patients results in altered inflammatory responses in the long term, supports the concept that an acute Q fever infection might be able to induce long‐term changes in monocytes, and therefore possibly also myeloid progenitor cells and macrophages. The concept of trained immunity is based on metabolic and epigenetic remodeling of monocytes, macrophages, and their progenitor cells, following insults such as infection, vaccination, or inflammatory diseases (e.g. atherosclerosis) 10, 11, 12, 13, 14. Epigenetic remodeling on promoter regions of pro‐inflammatory cytokines, e.g. TNF‐α and IL‐6, can result in transcriptionally active chromatin, often accompanied by higher expression of open chromatin marks such as H3K4me3 11, 22. Such remodeling can result in an increased inflammatory response upon a second, nonspecific, infectious stimulus. As a counterbalance of trained immunity, certain incentives are known to inflict long lasting decreased inflammatory responses, i.e. tolerance 22, 23, 24, 25. Such a process, inflicted by a strong initial incentive, e.g. acute Q fever infection, could perhaps explain the altered cytokine responses in these QFS patients and arises from similar metabolic and epigenetic remodeling.

We found that monocytes of QFS patients produced less cytokines than those of age‐ and sex‐matched healthy controls upon stimulation with various ligands. It could therefore be postulated that, in these patients, the acute *Coxiella* infection resulted in altered monocyte responsiveness through long‐lasting epigenetic, and metabolic, remodeling at the time of infection. Given the differing results in monocyte responsiveness when using various ligands, such remodeling is likely to be complex and vary per cytokine and chemokine promoter region. If monocytes of QFS patients are indeed transcriptionally altered upon encountering infections, e.g. upper respiratory tract viruses, it could be conceived that this leads to greater susceptibility to viral upper respiratory tract infections.

Since mRNA expression of IL‐1β and TNF‐α show a similar trend as cytokine production when comparing QFS patients to healthy controls, it is suggestive that the origin of the differences seen between these groups is regulated (pre‐)transcriptionally, e.g. through epigenetic remodeling.

Investigation of H3K4me3 on the promoter regions of IL‐1β, MCP‐1, IP‐10, IL‐10, and IL‐13 showed a decreased expression, suggestive for less transcriptionally active chromatin. This could in part explain the decreased mRNA expression and cytokine production in these QFS patients compared to their age‐ and sex‐matched healthy controls. However, it should be noted that this is an investigation of a very specific part and alteration on these promoter regions. Many more modifications are known to occur at these regions, which could all influence activation, inhibition, but also each other 23, 26. Perhaps these results could best serve as an indication that the immunologic alterations seen in QFS could in part be explained by epigenetic remodeling in myeloid cells. This epigenetic remodeling could very well be induced by acute infection with *C. burnetii*, and result in long‐lasting changes in monocytes, macrophages, and their progenitor cells. Future investigations on *Coxiella*‐induced epigenetic remodeling in these cells are of great interest but should employ a broader scope, for example by means of ChIP‐sequencing.

As the results of this study merely serve as an indication that epigenetic remodeling might contribute to the diminished immune response in QFS patients who report having more frequent and severe upper respiratory tract infections since their acute Q fever infection, a larger confirmatory study is warranted. We suggest employing ChIP‐sequencing and investigation of methylated DNA in a prospective longitudinal study in which QFS patients with more frequent and severe upper respiratory tract infections are compared with: (1) QFS patients without such complaints, (2) patients who recovered from the acute Q fever infection without persistent complaints, and (3) healthy controls, all matched for age and sex. In order to avoid recall bias, it would be of interest to objectify the amount and severity of upper respiratory tract infections in these patients.

## Conclusion

In conclusion, QFS patients who report having more frequent and severe upper respiratory tract infections since their acute Q fever infection showed diminished immune responses to various stimuli compared to age‐ and sex‐matched healthy controls, possibly due to epigenetic remodeling as a result from the acute Q fever infection. Future research on the role of *Coxiella*‐induced epigenetic changes in the innate immune cells of these patients and their role in the complaints they exhibit is warranted.

## Materials and Methods

### Study population

The study population consisted of 15 QFS patients who reported having more (≥3/year), more severe, and longer‐lasting upper respiratory tract infections since their acute Q fever infection. Upper respiratory tract infections were not documented. QFS patients were matched for age‐ and sex (±10 years) with 16 healthy controls.

All QFS patients protracted QFS during the Dutch Q fever outbreak between 2007 and 2011 and were diagnosed at the Radboud Expert Center for Q fever, Nijmegen, the Netherlands, after a uniform work‐up according to the Dutch guideline on QFS 27. All QFS patients met the following diagnostic criteria: (i) fatigue lasted ≥ 6 months; (ii) sudden onset of severe fatigue (defined as a score ≥ 35 on the subscale fatigue severity of the Checklist Individual Strength questionnaire), or significant increase in fatigue, both related to a symptomatic acute Q fever infection; (iii) chronic Q fever and other causes of fatigue, somatic or psychiatric, were excluded; and (iv) fatigue resulted in significant functional impairment (defined as a total score ≥450 on the Sickness Impact Profile‐8 questionnaire).

All healthy controls, i.e., colleagues from the department of Internal Medicine at the Radboud University Medical Center, Nijmegen, who lived in areas previously endemic for Q fever during the Dutch outbreak between 2007 and 2011, tested negative on Q fever serology (Immunofluorescence assay, or IFA; Focus Diagnostics, Cypress, CA, USA), and had a score <35 on the subscale fatigue severity of the Checklist Individual Strength questionnaire and a score <450 on the Sickness Impact Profile‐8 questionnaire.

### Monocyte isolation and stimulation

http://PBMC isolation was performed by dilution of blood in PBS (1:1) and fractions were separated by density centrifugation over Ficoll‐Paque (Ficoll‐Paque Plus; GE healthcare, Zeist, The Netherlands). Cells were washed three times with cold PBS and resuspended in RPMI 1640 Dutch modification culture medium (Life Technologies/ Invitrogen, Breda, the Netherlands) supplemented with 50 μg/mL gentamicin, 2 mM Glutamax, and 1 mM pyruvate (Life Technologies). http://Percoll isolation of http://monocytes was performed as previously described 28. Briefly, 150–200 × 10^6^ PBMCs were layered on top of a http://hyperosmotic Percoll solution (48.5% Percoll, 41.5% sterile H_2_O, 0.16 M filter sterilized NaCl) and centrifuged for 15 min at 580 × *g*. The interphase layer was isolated and cells were washed once with cold PBS. Cells were resuspended in culture medium as described above. A total of 1  ×  10^5^ monocytes were seeded per well on flat‐bottom 96‐well plates (Corning, New York, USA) and stimulated for 24 h with RPMI only as a negative control, LPS (Sigma–Aldrich, St. Louis, MO; from *E. coli*), or various viral ligands. After 24 h, plates were centrifuged and supernatants were stored at −20°C until cytokine assessment.

### Viral ligands

The following viral ligands were used in the experiments: 10 μg/ml Poly I:C (polyinosinic:polycytidylic acid; Invivogen, Toulouse, France), signaling through TLR‐3, 10 μg/mL R848 (Resiquimod; Invivogen, Toulouse, France) signaling through TLR7 and TLR8), and 1 μg/mL 5’ppp ds‐RNA (a ligand for the cytoplasmic retinoic acid‐inducible protein (RIG) receptor that senses viral RNA (Invivogen, Toulouse, France).

### Quantitative PCR

RNA was isolated using TRIzol reagent (Invitrogen) according to the protocol supplied by the manufacturer, and RNA was converted into cDNA using iScript cDNA synthesis kit (Biorad, Hercules, CA). Quantitative real‐time PCR (qPCR) was performed using power SYBR Green PCR master mix (Applied Biosystems, Carlsbad, CA) and the following primers: TNF‐α FWD‐5’‐AACGGAGCTGAACAATAGGC‐3’, REV‐5’‐TCTCGCCACTGAATAGTAGGG‐3’, IL‐1β FWD‐5’‐GCCCTAAACAGATGAAGTGCTC‐3’, REV‐5’‐GAACCAGCATCTTCCTCAG‐3’. PCR was performed using an Applied Biosystem StepOne PCR system using PCR conditions 2 min 50°C, 10 min 95°C, followed by 40 cycles at 95°C for 15 s, and 60°C for 1 min. RNA expression was corrected for differences in loading concentration using the signal of housekeeping protein GAPDH.

### Chromatin immunoprecipitation

For the assessment of histone methylation, ChIP was performed as described previously 29. In short, monocytes were cross‐linked in methanol free 1% formaldehyde (28908, Thermo Scientific), followed by sonication to shear‐DNA and IP using antibodies against H3K4me3 (Diagenode, Seraing, Belgium). ChIPed DNA was processed further for qPCR analysis. Primers used in the reaction are listed in Table S1. Samples were analyzed following a comparative Ct method, myoglobin was used as a negative control and H2B as a positive control for H3K4me3, according to the manufacturer's instructions.

### Determination of altered cytokine responses of QFS patients compared to healthy controls

By determining production of monocyte‐derived cytokines IL‐6, TNF‐α, and IL‐1β in monocytes of QFS patients compared to healthy controls upon stimulation with LPS and various viral ligands, we investigated whether a difference in monocyte reactivity could be observed between QFS patients with frequently recurring and severe upper respiratory tracts infections, and healthy controls.

### Cytokine and chemokine assays

Cytokine and chemokine production were determined in supernatants using commercial ELISA kits for IL‐1 β, TNF‐α (R&D systems, MN, USA), and IL‐6 (Sanquin, Amsterdam, the Netherlands), and by using Luminex technology (Thermo Fisher Scientific, Waltham, USA; Cytokine 25‐plex Human Panel), following the manufacturer's instructions. Cytokines and chemokines determined by Luminex assay are listed in Table S2.

### Histone modifications at cytokine promoter regions of QFS patients compared to healthy controls

We investigated whether epigenetic markers on cytokine promoter regions were different between patients and controls. Histone modifications at cytokine promoter regions have been associated with trained immunity 11, especially H3K4me3.

### Statistical analysis

Data were analyzed using Graphpad Prism (Graphpad Software Inc., version 5.03). The Mann–Whitney test was used to determine differences between groups. Statistical significance was attained if *p* < 0.05.

### Ethical statement

All participants provided written informed consent, and the study was approved by the Medical Ethical Review Committee of the Arnhem‐Nijmegen region.

## Conflict of interest

The authors declare no conflict of interest.

**Abbreviations**
H3K4me3: histone 3 lysine 4 trimethylation
IQR: interquartile range
Poly I:C: polyinosinic:polycytidylic acid
QFS: Q fever fatigue syndrome
qPCR: quantitative real‐time PCR

## Supporting information

Supplementary table 1. Primers used for various cytokines and chemokines
Supplementary table 2. Luminex assay determining 25 different cytokines and chemokines.
Supplementary Figure 1. Negative and positive control for H3K4me3
