# Supplementary material for: A possible link between recurrent upper respiratory tract infections and lower cytokine production in patients with Q fever fatigue syndrome
Source: Eur J Immunol. 2019 May 9;49(7):1015–22. doi: 10.1002/eji.201848012 (PMC7163623; doi:10.1002/eji.201848012)
Supplement: Supplementary file 1 — Supplementary table 1. Primers used for various cytokines and chemokines Supplementary table 2. Luminex assay determining 25 different cytokines and chemokines. Supplementary Figure 1. Negative and positive control for H3K4me3 [file EJI-49-1015-s001.pdf]

European Journal of Immunology

Supporting Information
for
DOI 10.1002/eji.201848012

Ruud P.H. Raijmakers, Anne F.M. Jansen, Stephan P. Keijmel, Jos W.M. van der Meer, Leo A.B. Joosten, Mihai G. Netea and Chantal P. Bleeker-Rovers

A possible link between recurrent upper respiratory tract infections and lower cytokine production in patients with Q fever fatigue syndrome

© 2019 The Authors. *European Journal of Immunology* published by WILEY-VCH Verlag GmbH & Co. KGaA, Weinheim.

Supplementary table 1. Primers used for various cytokines and chemokines

Cytokine/chemokine	Primer	Forward	Reverse
**IL-1β**	1	AATCCCAGAGCAGCCTGTTG	AACAGCGAGGGAGAAACTGG
	2	CATGGCTGCTTCAGACACCT	ACACATGAACGTAGCCGTCA
	3	TGAGTGACTTCCCCATGACG	TCTGCTCCAGCTCTCCTAGC
	4	CTGGCGAGCTCAGGTACTTC	ACACATGAACGTAGCCGTCA
	5	TCAGATGAGGTCCAAAGCGG	TTTGCTACCTGGGGTGAAGC
**MCP-1**	1	CCGAGAGGCTGAGACTAACC	CTATGAGCAGCAGGCACAGA
	2	CCCAGAAACATCCAATTCTCA	GGAATGAAGGTGGCTGCTAT
**IP-10**	1	ATGCAGGTACAGCGTACAGTT	GAACACTGTGCTAACCTTCTCTG
**IL-10**	1	ATGCGGTCTTTTTGATGCCC	GGTCCCATCACTGTTGAATCCT
	2	TCCAGCTGATCCTTCATTTGCT	AAAGGGCATCAAAAAGACCGC
**IL-13**	1	CATTGCTCTCACTTGCCTTGG	CCGACACTCACCTTCTGGTT
	2	GGTCAACATCACCCAGAACCA	TCATCTTGGGAATCACCCACC
	3	TGGTCAACATCACCCAGAACC	GCTCATGACCTCATCTTGGGA

Abbreviations: *IL* = interleukin; *MCP* = monocyte chemoattractant protein; *IP* = Interferon Gamma-Induced Protein.

Supplementary table 2. Luminex assay determining 25 different cytokines and chemokines.

Luminex	Cytokines	Chemokines
**25-plex assay panel**	GM-CSF	Eotaxin
	IFNα	IP-10
	IFNγ	MCP-1
	IL-1β	MIG
	IL-1RA	MIP-1α
	IL-2	MIP-1β
	IL-2R	RANTES
	IL-4	
	IL-5	
	IL-6	
	IL-7	
	IL-8	
	IL-10	
	IL-12 (p40/p70)	
	IL-13	
	IL-15	
	IL-17	
	TNFα	

Abbreviations: *GM-CSF* = granulocyte-macrophage colony–stimulating factor; *IFN* = interferon; *IL* = interleukin; *TNF* = tumor necrosis factor; *IP* = Interferon Gamma-Induced Protein; *MCP* = monocyte chemoattractant protein; *MIG* = monokine induced by gamma interferon; *MIP* = macrophage inflammatory protein; *RANTES* = regulated on activation, normal T cell expressed and secreted.

Supplementary Figure 1. Negative and positive control for H3K4me3

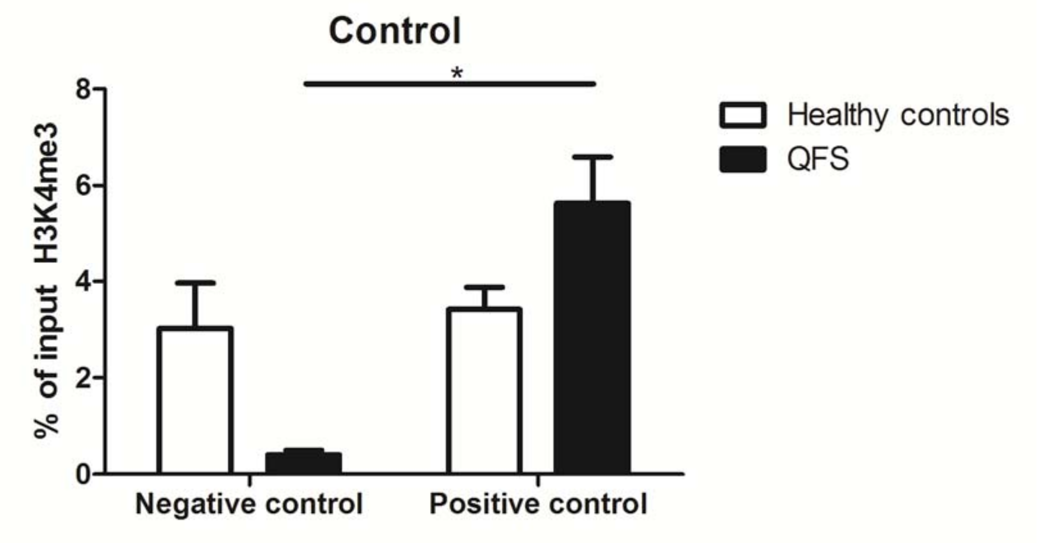

Expression of H3K4me3 in monocytes of QFS patients compared to healthy controls, using myoglobin as a negative and H2B as a positive control. Results are depicted as mean ± SEM. Data are derived from one single experiment that consisted of 15 patients and 16 healthy controls.

Abbreviations: *H3K4me3* = histone 3 lysine 4 trimethylation; *QFS* = Q fever fatigue syndrome; *SEM* = standard error of mean.

* $P \leq 0.05$
